# Amiodarone-Induced Lung Toxicity: A Case Initially Not Correctly Framed

**DOI:** 10.7759/cureus.36818

**Published:** 2023-03-28

**Authors:** Marco Umberto Scaramozzino, Giovanni Sapone, Ubaldo Romeo Plastina, Mariacarmela Nucara

**Affiliations:** 1 Department of Pulmonology, Ambulatory "La Madonnina" Reggio Calabria, Reggio Calabria, ITA; 2 Department of Cardiology, Polyclinic Madonna della Consolazione, Reggio Calabria, ITA; 3 Department of Radiology, Ecorad LTD Study of Radiology and Ultrasound, Reggio Calabria, ITA; 4 Department of Clinical and Experimental Medicine, Section of Cardiology, University of Messina, Messina, ITA

**Keywords:** ground glass opacity, ocs, hyper-reactivity, amiodarone, aipt

## Abstract

Amiodarone-induced pulmonary toxicity (AIPT) is one of the most serious adverse effects of amiodarone and is one of the leading causes of death associated with its use. The onset of AIPT depends on dosage, patient’s age, and pre-existing pulmonary pathologies; typically, the adverse effects stop progressing when a cumulative dose higher than 150 mg is reached. The risk of developing amiodarone-induced pulmonary fibrosis is directly related to the dosage and duration of administration. In this case report, the effect of a prolonged overdose of amiodarone taken at doses of 200 mg/day for two years is reported, with symptoms and instrumental evidence of respiratory pathology induced by amiodarone drug toxicity. Comorbidities, oxygen therapy, invasive procedures, and surgical interventions can trigger pulmonary symptoms. Despite significant advances in understanding AIPT, its etiology and pathogenesis remain poorly understood. The role of steroids in the treatment of AIPT is still under debate as most reports of improvement after amiodarone withdrawal differ little from those in which concomitant steroid therapy was used. In clinical practice, therapeutic doses of corticosteroids may be indicated for patients with AIPT; usually, a starting dose of prednisone from 40 to 60 mg daily, which is then gradually reduced, is prescribed. The pharmacodynamics of amiodarone determines a treatment period of four to 12 months.

The patient with AIPT in this case report, who markedly improved after treatment with prednisone at a starting dose of 50 mg/day, which was then gradually tapered. At the end of the therapy, the computed tomography (CT) scan revealed the disappearance of most of the scattered ground-glass opacities and of the thickening indicating bi-apical pulmonary fibrosis.

The case report is unique because:

1) Bronchoalveolar lavage (BAL)/transbronchial biopsy was not used for diagnosis.

2) The case was framed based on the patient's laboratory and clinical data.

3) The pathology is normally prevalent in men rather than women.

## Introduction

Amiodarone is an effective antiarrhythmic often used in the perioperative period after cardiac surgery; however, it can have significant adverse effects. After the first dose, amiodarone reaches its peak plasma concentration within three to seven hours; the onset of action ranges from a few days to a few weeks. Liver diseases, interactions with inhibitors or inducers of the cytochrome P450, and the patient’s age influence its bioavailability. Due to their high lipophilicity, high concentrations of amiodarone and its metabolites can be found in fat and well-perfused organs, such as the liver, lungs, and skin, where they interact with the phospholipid metabolism. The accumulation of amiodarone can cause corneal deposits and photophobia, peripheral neuropathy, thyroid disorders, hepatitis, dyspepsia, and photosensitivity. Pulmonary complications account for only 4 to 6% of all complications but they have the most significant impact, as they can be fatal [1].

Amiodarone-induced pulmonary toxicity (AIPT) is classified into acute, subacute, and chronic [2]. It can develop within the first one to one and a half years after the start of treatment, and it occurs quickly in patients taking high doses of amiodarone. Signs and symptoms of AIPT are non-specific: general malaise, dry cough, pleuritic chest pain, and progressive dyspnea, which is enhanced if the drug also induces hyperthyroidism. Amiodarone can cause different lung injuries, such as organizing pneumonia, pleural disease, chronic interstitial pneumonitis, diffuse alveolar damage, pulmonary hemorrhage, and lung nodules [3]. Amiodarone also affects the mechanism of action of other drugs, such as anticoagulants (i.e., warfarin), statins (i.e., simvastatin and atorvastatin), and antiretrovirals for HIV patients.

The drug has a dose-dependent negative inotropic effect that decreases systemic vascular resistance. However, the dosage that is usually prescribed (between 200 and 600 mg daily) has minimal impact on hemodynamics [4].

The diagnosis of AIPT can be made if there are clinical manifestations in the lungs, thyroid, liver, and eye. Laboratory tests may show increased erythrocyte sedimentation rate (ESR), leukocytosis, increased lactate dehydrogenase (LDH), and peripheral eosinophilia. Computed tomography (CT) of the chest can show alveolar, interstitial, or mixed alveolar-interstitial ground-glass opacities [5]; asymmetric or bilateral lung involvement; reticular and perilobular interstitial opacities; basal traction bronchiectasis of one or more subpleural nodules (6-12%); pleural thickening (with pleuritic chest pain or coughing during physical examination); sometimes dense, bibasilar, and reticular opacities that cause coarse crackles on chest auscultation. Patients usually have significant hypoxemia and sometimes weight loss. Lung function tests reveal in most cases a specific condition characterized by a reduced diffusing capacity of the lungs for carbon monoxide (DLCO), which is even lower in patients with pre-existing lung disease, such as chronic obstructive pulmonary disease (COPD) [6]. In this case, the patient was unable to perform the DLCO at the first visit because she had a functional inability to perform a forced inhalation and hold her breath for 10 seconds. It was therefore not included as the machine failed the three tests performed.

## Case presentation

**Figure 1:**
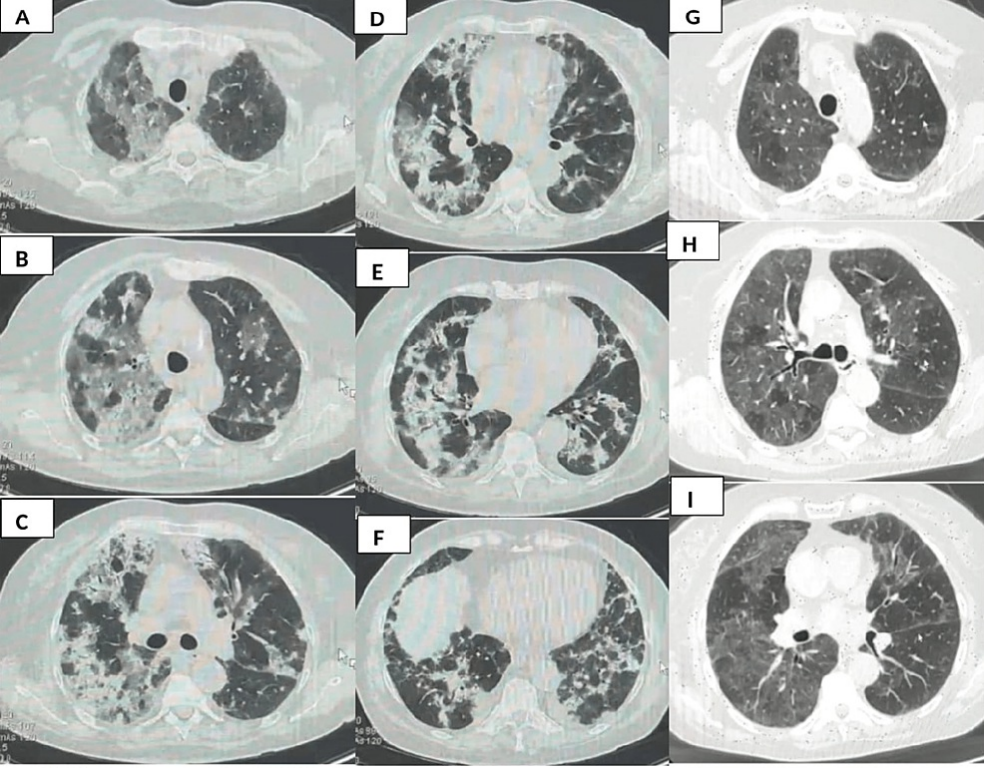
A: Time zero chest CT scan showing areas of bilateral apical centrilobular consolidation with thickening of the pulmonary reticular interstitium of the intra- and interlobular septa, with predominance in the right apical areas. B: Time zero chest CT scan showing areas of bilateral apical centrilobular consolidation with thickening of the pulmonary reticular interstitium of the intra- and interlobular septa, with predominance at the right apical zones with presence in both lungs at the submantellar zones of solid centrilobular consolidations. C: Chest CT scan at time zero showing areas of right perilobular consolidation with thickening of the reticular pulmonary interstitium of the intra- and interlobular septa and prevalence in the right middle field of areas of solid centrilobular consolidation. D-E-F: Time-zero chest CT scan showing areas of peri- and mid-basal centrilobular consolidation prevailing on the right, thickening of the reticular pulmonary interstitium intra- and interlobular septa, and predominance in the mid-right field of areas of solid centrilobular consolidation. G-H-I: Chest CT scan carried out at three months of therapy with an oral corticosteroid, inhaled corticosteroid, and a long-acting bronchodilator, showing apical, middle, and basal areas almost complete regression of the areas of consolidation. CT: computed tomography

A 60-year-old caucasian female former smoker of 10 pack/year presented to the emergency department for dyspnea and evening febrile episodes in December 2022. The patient had a history of esophageal hiatal hernia, surgery for a cerebral aneurysm, past SARS-CoV-2 infection in July 2022, and arrhythmic heart disease for which she had been taking amiodarone at a dosage of 200 mg/day for the last two years. The patient declares no exposure to chemicals, vaping, pets including birds, hot tubs, or humidifiers. On hospital admission, the patient had normal vital parameters blood pressure: 120/80 mmHg, SpO2: 93% peripheral oxygen saturation, and heartbeat: 90 beats per minute. On thoracic examination, she showed reduced vesicular murmur with bilateral "Velcro-like" crackles, bilateral hypo-transmitted tactile fremitus, and clear pulmonary sound. The chest CT scan (Figures 1A-F) showed the presence of multiple alternating pleura thickenings with ground-glass opacities that were initially thought to be the result of COVID-19 pneumonia. The echocardiography revealed a mild mitral valve insufficiency and a cardiac ejection fraction of 62%. The thyroid ultrasound showed diffuse nodular formations, some cysts, and an enlargement of the thyroid gland. Neither bronchoalveolar lavages (BALs) nor transbronchial biopsies nor bronchoscopy was carried out during her hospitalization, as she was discharged against the advice of the medical staff by patient and because the facility where she had been admitted did not have the possibility of carrying out the endoscopic procedure due to a lack of instrumentation. The spirometry showed a moderate restrictive ventilatory defect. The results of the spirometry and the blood test are summarized in Table 1.

**Table 1:** Spirometry and laboratory tests which indicate the baseline values at the first examination. FEV1%: Percentage of predicted value of FEV1 FVC%: Percentage of predicted value of FVC FEV1: Maximum Expiratory Volume at first second FVC: Forced vital capacity TLC: total lung capacity CRP: C-reactive protein WBC: white blood cell count Cells x mm^3^: cells per cubic millimeter LDH: Lactic dehydrogenase FEF25-75%: forced expiratory flow between 25 and 75% of FVC ESR: Erythrocyte sedimentation rate SARS-CoV-2: severe acute respiratory syndrome coronavirus 2 PCR: polymerase chain reaction PEF: Peak expiratory flow mm/h: millimeters per hour mU/mL: milliUnits/milliliter µU/mL: picoUnit/milliliter ng/mL: nanograms/milliliter mg/L: milligrams/liter U.I./mL: International Unit/milliliter

Parameter (units)	Value
FVC%	79% predicted
FEV1%	100% predicted
FEV1/FVC% ratio	125% predicted
FEF_25-75%_	242% predicted
TLC%	56% predicted
PEF%	78% predicted
ESR	120 mm/h
CRP	13.9 mg/L
LDH	683 mU/mL
Fecal occult blood	Positive
TSH	12 µU/mL
Antithyroglobulin antibodies	107 U.I./mL
25-OH Vitamin D	17.9 ng/mL
WBC	11,850 cells mm^3^
Neutrophils	7,160 cells mm^3^
Eosinophils	1,230 cells mm^3^
SARS-COV-2 nasopharyngeal swab PCR	Negative

On discharge from the hospital, she stopped taking amiodarone and was prescribed prednisone 25 mg twice daily for 20 days [7]; the corticosteroid was then tapered as follows: 12.5 mg twice daily for 20 days, 6.25 mg twice daily for 30 days, 6.25 mg once daily until the next clinical and spirometry check at three months. The patient was also given antibiotic therapy with quinolone and macrolide for eight and six days, respectively for suspicion of bacterial superinfection. The blood gas analysis performed showed no hypoxemia.

At the three-month check-up, the patient is found to be in an improved clinical condition with improvement in dyspnoea and a marked reduction in auscultatory crepitations at the previous thoracic objective examination. SpO2: 97% in ambient air, heart rate: 80 beats per minute. No change in heart rate after discontinuation of amiodarone as the attending cardiologist replaced the therapy with a selective beta blocker. The functional test measurements improved, although a mild restrictive deficit persisted and the peak expiratory flow (PEF) increased by 45%, indicating bronchial hyperreactivity. Chest CT showed significant improvement in the apical, middle, and basal areas that almost complete regression of the areas of consolidation and remaining thickening of the reticular pulmonary interstitium of the intra- and interlobular septa consistent with non-specific interstitial pneumonia (Figures 1G-H). There were extensive areas of ground-glass thickening that were mainly centrilobular and involved the parenchyma of both lungs, the right lung was more affected with some small lymph nodes in the mediastinum (14 mm maximum diameter). These findings indicated a significant improvement compared to the first examination. The results of the blood test and spirometry at three months are shown in Table 2.

**Table 2:** Spirometry and laboratory test values which indicate the results at three months after treatment with oral corticosteroids and inhaled corticosteroids/long-acting-beta2-agonists (ICS/LABA) twice daily at the second examination. FEV1%: Percentage of predicted value of FEV1 FVC%: Percentage of predicted value of FVC FEV1: Maximum Expiratory Volume at first second FVC: Forced vital capacity TLC: total lung capacity PCR: C-reactive protein WBC: white blood cell count Cells x mm3: cells per cubic millimeter LDH: Lactic dehydrogenase FEF25-75%: forced expiratory flow between 25 and 75% of FVC VES: Erythrocyte sedimentation rate SARS-CoV-2: severe acute respiratory syndrome coronavirus 2 PCR: polymerase chain reaction PEF: Peak expiratory flow mm/h: millimeters per hour mU/mL: milliUnits/milliliter µU/mL: picoUnit/milliliter ng/mL: nanograms/milliliter mg/L: milligrams/liter U.I./mL: International Unit/milliliter

Parameter (units)	Value
FVC%	88% predicted
FEV1%	73% predicted
FEV1/FVC% ratio	120% predicted
FEF_25-75%_	201% predicted
TLC%	74% predicted
PEF%	123% predicted
ESR	115 mm/h
CRP	2 mg/L
LDH	468 mU/mL
Fecal occult blood	Negative
TSH	10 µU/mL
Antithyroglobulin antibodies	102 U.I./mL
Vitamin D	15 ng/mL
WBC	8,000 cells mm^3^
Neutrophils	5,160 cells mm^3^
Eosinophils	230 cells mm^3^
SARS-CoV-2 nasopharyngeal swab PCR	Negative

## Discussion

Amiodarone is a very effective and commonly used antiarrhythmic drug, however, different studies have underlined its numerous adverse effects, among which the most dangerous is pulmonary toxicity [8]. The dosage of amiodarone, duration of the treatment, patient’s age, and pre-existing pulmonary pathologies influence the onset of AIPT. Lung injuries are caused by the accumulation of phospholipid complexes in histocytes and type II pneumocytes, the medication can lead to mild to severe lung injuries, involving both the parenchyma and the pleura [9], and may cause respiratory failure. Reducing the dosage of amiodarone considerably decreases the incidence of these complications, however, AIPT can be life-threatening and there can be acute cases [10].

The diagnosis of AIPT is typically done with a differential diagnosis by comparing the symptoms of other interstitial lung diseases, left ventricular failure, and infectious diseases [11]. In approximately 45% of patients, pulmonary function tests highlight a moderately restrictive pattern on spirometry with a lower forced vital capacity (FVC) and a small reduction in the diffusing capacity for carbon monoxide (DLCO) [12]. Blood tests can suggest the presence of a nonspecific inflammatory syndrome, showing mild leukocytosis and higher values of erythrocyte sedimentation rate and C-reactive protein (CRP); however, these are nonspecific markers that identify pulmonary inflammation [13].

Even though the adverse effects of amiodarone are well-documented, physicians and patients do not often follow the guidelines for monitoring the therapy. For instance, the serum levels of amiodarone, which can be measured in specific medical centers and indicate toxicity if above 2.5 mg/L, are not regularly measured [14].

The entire team that interacts with the patient is responsible for preventing the adverse effects of amiodarone, from the specialist to the general practitioner to the pharmacist [15]; they can also implement a multidisciplinary approach that helps improve the quality of life and health outcomes of the patient [16]. Both the medical team and the patient need to take responsibility for the follow-ups that are needed to monitor the therapy with amiodarone. Moreover, up-to-date information and communication between patients and doctors are needed to improve the clinical condition of patients with AIPT.

The other consideration to be made in this clinical case concerns the scarcity of human and instrumental resources in public hospitals, which increasingly directs patients to turn to private specialists and allows them to see cases that are normally dealt with in a hospital setting. The territorial specialist is therefore required to find out about the systemic side effects of individual drugs, which makes it possible to manage the patient without subjecting him or her to potentially invasive procedures.

## Conclusions

The continuous steroid therapy improved the clinical and functional parameters of the patient. Diagnosing AIPT is difficult and usually done by differential diagnosis to exclude idiopathic pulmonary fibrosis or fibrosis caused by an infection; it is based on clinical manifestations of respiratory insufficiency and on the results of a chest CT scan that reveals the presence of interstitial lung disease, sometimes even of usual interstitial pneumonia (UIP). Early recognition of respiratory complications induced by amiodarone followed by a targeted therapy is essential for a favorable clinical evolution. If there is clinical suspicion and amiodarone is not discontinued immediately, the consequences on the patient’s health can be serious. In this case, the replacement of amiodarone with a selective beta-blocker drug was, together with the addition of systemic and inhaled corticosteroids, decisive in resolving the case. The role of steroids in the management of AIPT is still controversial. However, it has been shown that patients who developed drug toxicity but had to keep intaking amiodarone at the same or lower doses for their arrhythmia improved with the addition of steroids. Thus, corticosteroids may be indicated for the treatment of AIPT in some cases. However, the dosage and duration of the therapy with steroids have not been established yet. Prednisone is usually started at doses of 40 to 60 mg/day orally with progressive tapering. The pharmacodynamics of amiodarone determines a prolonged treatment from four to 12 months.
